# Multi-step planning of eye movements in visual search

**DOI:** 10.1038/s41598-018-37536-0

**Published:** 2019-01-15

**Authors:** David Hoppe, Constantin A. Rothkopf

**Affiliations:** 1 Department of Psychology, Technical University Darmstadt, Darmstadt, Hesse Germany; 2 Centre for Cognitive Science, Technical University Darmstadt, Darmstadt, Hesse Germany; 3 Frankfurt Institute for Advanced Studies, Goethe University, Frankfurt, Hesse Germany

## Abstract

The capability of directing gaze to relevant parts in the environment is crucial for our survival. Computational models have proposed quantitative accounts of human gaze selection in a range of visual search tasks. Initially, models suggested that gaze is directed to the locations in a visual scene at which some criterion such as the probability of target location, the reduction of uncertainty or the maximization of reward appear to be maximal. But subsequent studies established, that in some tasks humans instead direct their gaze to locations, such that after the single next look the criterion is expected to become maximal. However, in tasks going beyond a single action, the entire action sequence may determine future rewards thereby necessitating planning beyond a single next gaze shift. While previous empirical studies have suggested that human gaze sequences are planned, quantitative evidence for whether the human visual system is capable of finding optimal eye movement sequences according to probabilistic planning is missing. Here we employ a series of computational models to investigate whether humans are capable of looking ahead more than the next single eye movement. We found clear evidence that subjects’ behavior was better explained by the model of a planning observer compared to a myopic, greedy observer, which selects only a single saccade at a time. In particular, the location of our subjects’ first fixation differed depending on the stimulus and the time available for the search, which was well predicted quantitatively by a probabilistic planning model. Overall, our results are the first evidence that the human visual system’s gaze selection agrees with optimal planning under uncertainty.

## Introduction

Actively deciding where to direct our eyes is an essential ability in fundamental tasks, which rely on acquiring visual information for survival such as gathering food, avoiding predators, making tools, and social interaction. As we can only perceive a small proportion of our surroundings at any moment in time due to the spatial distribution of our retinal receptor cells^1^, we are constantly forced to actively target our visual apparatus towards relevant parts of the visual scene using eye movements^2^. Thus, vision is a sequential process of active decisions^3^. Perceptually, these decisions have been characterized in terms of targeting gaze towards locations that are most salient^4^, maximizing knowledge about the environment^5–7^, or optimizing performance in the ongoing task^8–12^. Much less research has investigated, how the visual system selects sequential decisions in these tasks.

The sequential nature of eye movements raises the question, how each subsequent action is selected to achieve the task goal. This question can only be answered quantitatively with reference to a computational model. Several models have been proposed to describe possible strategies differing with respect to how future rewards influence the selection of the next action. The most naive strategy suggests that the visual system selects the location at which the task relevant criterion such as information about the search target or immediate reward is maximal^4–7,13^. This corresponds to always moving to the location, which currently is believed to be the most likely location of the target. E.g., saliency models posit that the visual system maintains an internal relevance map and that the next saccade moves gaze to the location currently having highest saliency^4^. Similarly, the ‘maximum-a-posteriori-searcher’^13,14^ moves to the location with the highest probability of containing the target. Empirical studies have found partial support for humans adopting this strategy in a number of tasks^4–7^. A more sophisticated strategy has been proposed by a number of other models, according to which the visual system selects a target, such that the criterion of the search task is expected to be maximal *after* having carried out the single next gaze shift^8–10,12^. E.g., the ‘ideal-searcher’ by Najemnik and Geisler^8^ will saccade in between two potential targets, which helps maximally in deciding, which one of the two potential targets is the correct target.

For tasks that require only a single action, the ‘ideal searcher’^8^ behaves optimally. However, many real world tasks involve more than a single isolated action. For sequences of eye movements, delayed rewards obtained only after a sequence of actions can play a crucial role. Thus, what strategy should the visual system employ to select several actions in sequence? The answer to this question leads to the third strategy, which is readily available within the artificial intelligence, machine learning, optimal control, and reinforcement learning literature^15–17^: the optimal sequence of actions in general involves planning. Behavioral sequences are planned when “deciding on a course of action by considering possible future situations before they are actually experienced” (p. 9 of ref.^16^). Hence, planning is defined as taking future rewards into consideration during current action selection. In contrast, a policy is called “myopic” or “greedy”, if only the immediate reward is taken into account (p. 632 in ref.^15^).

A classic example from the optimal control and reinforcement learning literature is the mountain car problem, in which a car is located in the valley between two hills. Possible actions for the driver are to accelerate forward or backward and the goal is to reach the top of one of the mountains. However, the mountain is too steep to conquer from the valley. Instead, momentum has to be built by going in the opposite direction first. So, the car first has to move *away* from the target to build up momentum to later reach it^16^. Hence, the next action associated with the maximum immediate reward *r*_0_ (‘get closer to the target’) is not necessarily the action that yields the maximum reward for the whole action sequence *r*_0_ + *r*_1_ + $\cdots$ + *r*_*n*_. As a consequence, optimal action selection for sequential behavior depends on the horizon *n*, i.e., the number of future rewards that are incorporated into the selection of the next action. Thus, if two actions are considered, the horizon is two and planning needs to consider the outcome of the first and second action to select both decisions.

Surprisingly, all of the reviewed computational models for eye movement selection are myopic, i.e. they choose actions that maximize the immediate reward^8,10–14,18,19^, either by moving gaze to the currently most likely target or to the target that promises to reveal the most likely target after a single next eye movement. In this case, the horizon equals to one as only the next reward is used for action selection. In practice, the problem of delayed rewards is circumvented by either investigating only single saccades or by choosing tasks where both policies, myopic and planned, may lead to similar solutions. To our knowledge, there exist neither computational models nor empirical data investigating whether humans are capable of planning eye movements. This is even more surprising considering the results of behavioral investigations which have interpreted a variety of empirical findings as evidence for human gaze planning^20–23^. These studies have shown that the latency of the first saccade was higher for longer sequences of saccades^21^. Also, discrimination performance was enhanced at multiple locations within an instructed sequence of saccades^22^. Furthermore, if an eye movement sequence was interrupted by additional information midway the execution of the second saccade was delayed^23^. While these results suggest that a scanpath of at least two saccades is internally prepared before execution, it is unclear whether multiple future fixation locations are jointly chosen to maximize performance in a task, which is a computational signature of planning.

In the present study, we devised computational models for the three search strategies described above, i.e selecting the location of highest target probability (the ‘maximum-a-posteriori searcher’^13,14^), selecting the target that will lead to best disambiguation after the next gaze shift (ideal-observer based searcher^8^), and selecting a sequence of gaze targets to maximize overall task performance (a probabilistic planning based searcher). All three strategies were formalized within the framework of partially observable Markov decision processes^15–17^. Using these models, we implemented a visual search task to investigate, whether the human visual system is capable of planning. Because myopic policies such as the ‘maximum-a-posteriori searcher’^13^ or the ‘ideal searcher’^8^ choose the next gaze target only based on the immediate reward while ignoring future rewards, actions selected by these models do not depend on the length of the entire action sequence. By contrast, an action within a planned policy depends on the entire sequence^15–17^. This fundamental difference can be used to derive an experimental design for testing the planning capabilities of the visual system. We formalized these three strategies within the framework of partially observable Markov decision processes^15–17^ and then derived algorithms for all three strategies. These models were employed to generate and select stimuli, for which the models predict a significant difference in strategies, such that the two potential models (planned or myopic) led to different gaze sequences. Crucially, we also selected stimuli for which the myopic and planning strategies did not differ substantially. The reason is, that this should not only further validate the proposed planning model but also reconcile the present study with previous empirical investigations, which had found evidence that human gaze strategies are well described by an ‘ideal searcher’ in some tasks. Using behavioral analyses and Bayesian model comparison we found strong evidence that the human visual system is capable of planning gaze sequences.

## Results

**Figure 1:**
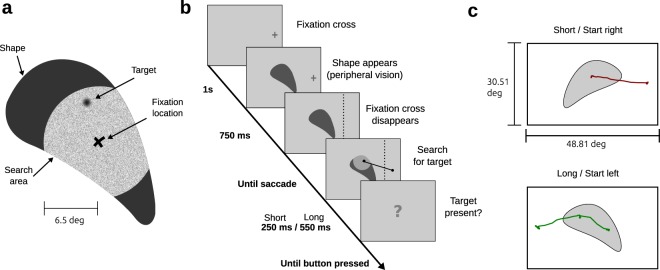
Experimental design. (**a**) Gaze contingent visual search paradigm. Targets were only visible in close proximity to the current fixation location (i.e., inside the search area). (**b**) Procedure for a single trial. Subjects fixated a fixation cross either shown on the left or the right side, respectively. After 1 s the shape was shown in the center of the screen, thus subjects were given access to peripheral information about the shape. Shapes were mirrored if necessary yielding equal distances for left and right starting points. After 750 ms the fixation cross disappeared and participants could initiate the search for the target. The search time was initiated by the participants’ gaze crossing the dotted line. The line, however, was not visible to the subjects. After the search interval was over the shape disappeared and participants were asked, whether it contained a target. Depending on the condition (short or long) subjects were able to perform one or two fixations inside the shape. (**c**) Raw gaze data is shown for a trial with short search time and initial fixation on the right side (upper panel) and for a trial with long search time and initial fixation on the left side (lower panel). Shapes were mirrored in a counterbalanced design to ensure equal orientation with respect to the initial fixation cross.

In our task, subjects searched for a hidden target within irregularly bounded shapes (Fig. 1a). Using a gaze contingent paradigm the hidden target only became visible if a fixation landed close enough. This search area was made explicit by showing the shape’s texture for all points closer than 6.5° to the fixation location. If a target was located in the search area, it became visible to the participant after a delay of 130 ms. Targets were easily detectable once they became visible (detection proportion: 98.2%). Overall, all shapes contained a target in half the trials, respectively. We used two durations as search intervals: a short interval (250 ms) providing enough time for a single saccade and a long interval (550 ms) providing enough time for two saccades. Trials were presented in blocks either containing only short intervals or long intervals, respectively. The procedure for a single trial is shown in Fig. 1b. By using a blocked design of 100 consecutive trials with the same interval length subjects knew about the upcoming trial duration (short or long).

### Computational models for action selection in visual search

Given the observer has formed a belief about the location of the target in the visual search task, how should gaze targets be selected? We derived models for the myopic observer *π*_*myopic*_ and the planning observer *π*_*planned*_ for our visual search task based on the framework of partially observable Markov decision processes (see Methods). In our experiment, participants directed their gaze to suitable locations within a shape. Subsequently, they indicated whether the shape contained a target or not through a button press. The quality of this decision depends on the fixated locations and improves if the fixation locations are chosen strategically to cover more area. Also, the probability of making a correct statement is proportional to the probability of finding the target. Depending on the search time, the action sequence in our task comprised one ((*x*_1_, *y*_1_); short condition) or two ((*x*_1_, *y*_1_, *x*_2_, *y*_2_); long condition) fixation locations.

**Figure 2:**
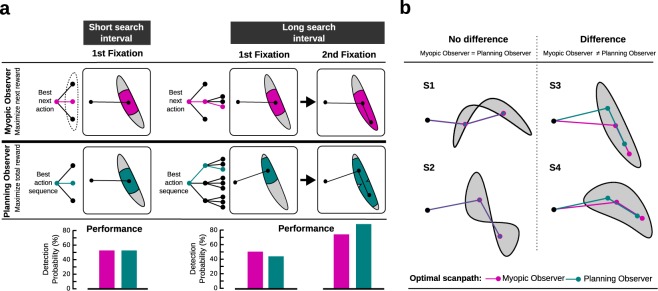
Computational models for visual search. (**a**) Illustration of optimal scanpaths for both models depending on the search time. For the short search interval (left side, one fixation) both models show the same behavior. For the long search interval (right side, two fixations), the myopic observer and the planning observer differ with respect to the scanpath. While the myopic observer’s next fixation is chosen to maximize the immediate reward (better performance after the first fixation, bottom row), the planning observer’s scanpath is chosen to maximize performance after two fixations (see also Supplementary Fig. 6). Computational complexity (depicted as decision trees) is higher for the planning observer as in the condition with long search intervals all two-fixation sequences are evaluated in order to maximize performance. Note that the second action in the long search interval is only necessary, if the target was not detected after the first saccade. (**b**) Shapes used in our visual search experiment. For each shape the optimal policy is shown for the myopic observer (pink) and the planning observer (green). Whether these models lead to different strategies depends on the particular shape. Scanpaths are the same for Shapes S1 and S2, but differ for S3 and S4.

For the short condition, a single fixation location (*x*_1_, *y*_1_) was selected. In this case, both strategies lead to the same action, because for both models only the consequences of a single gaze shift need to be taken into account. Hence, the maximal horizon of the sequence is 1, leading to:

1 $${\pi }_{{\rm{myopic}}}={\pi }_{{\rm{planned}}}=\mathop{{\rm{argmax}}}\limits_{({x}_{1},{y}_{1})}\,P\,({\rm{correct}}|{x}_{1},{y}_{1})$$

where *P*(correct| *x*_1_, *y*_1_) is the probability of finding the target when fixating (*x*_1_, *y*_1_), which is proportional to the amount of the shape covered by the search area (see Methods, for how this is computed). The action selection for the short search interval is depicted in the left panel of Fig. 2a.

For the long condition, a sequence of two fixation locations (*x*_1_, *y*_1_, *x*_2_, *y*_2_) was chosen resulting in a maximum horizon of two. In this case, the two strategies differ. First, the myopic observer uses the uncertainty of the current observation to select only the next gaze target such that the probability of detecting the location of the target will be maximal after each single saccade. Thus, the myopic observer sequentially chooses the fixation location with the maximum expected immediate reward resulting in the policy:

2 $${\pi }_{{\rm{myopic}}}:=(\mathop{{\rm{argmax}}}\limits_{({x}_{1},{y}_{1})}\,P({\rm{correct}}|{x}_{1},{y}_{1}),\mathop{{\rm{argmax}}}\limits_{({x}_{2},{y}_{2})}\,P({\rm{correct}}|{x}_{1},{y}_{1},{x}_{2},{y}_{2}))$$

where *x*_*n*_, *y*_*n*_ are the coordinates of *n*th fixation location and *P*(correct | *x*_*n*_, *y*_*n*_) denotes the probability of deciding correctly whether a target is present after the *n*th fixation.

By contrast, the planning observer uses the uncertainty of the current observation to select the upcoming gaze targets such that the probability of detecting the location of the target is expected to be maximal after the sequence of two saccades. Thus, the planning observer incorporates the whole sequence in the selection of all actions:

3 $${\pi }_{{\rm{planned}}}:=(\mathop{{\rm{argmax}}}\limits_{({x}_{1},{y}_{1})}\,P({\rm{correct}}|{x}_{1},{y}_{1},{x}_{2},{y}_{2}),\mathop{{\rm{argmax}}}\limits_{({x}_{2},{y}_{2})}\,P({\rm{correct}}|{x}_{1},{y}_{1},{x}_{2},{y}_{2}))$$

where *P*(correct | *x*_1_, *y*_1_, *x*_2_, *y*_2_) is the probability of a correct decision when fixating location (*x*_1_, *y*_1_) followed by (*x*_2_, *y*_2_).

Figure 2 illustrates the difference between the two computational strategies for the two conditions, i.e. the short search interval, allowing a single gaze shift, and the long search interval, allowing a maximum of two gaze shifts. The action selection for both the myopic and the planning observer for the long condition is shown in the right panel of Fig. 2a. Accordingly, three testable hypothesis can be derived from the computational models: *H*_1_: If eye movements are planned, we expect a difference in the location of the first fixation depending on the search interval for some stimuli. *H*_2_: We expect fixation locations to be better explained by the planning observer compared to the myopic observer. *H*_3_: The differences between the myopic and the planning observers also depend on the search shape, such that the gaze targets may coincide for the two models (Fig. 2b).

### Behavioral and model results

**Figure 3:**
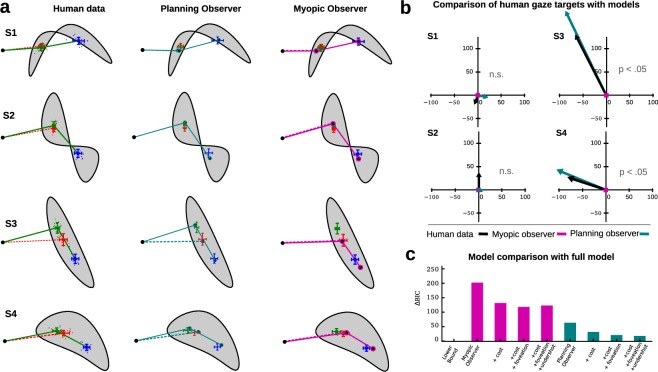
Main behavioral and model results. (**a**) Mean human scanpaths for both conditions (solid lines correspond to long search intervals, dashed lines correspond to short search intervals) are shown in the left column. Colors refer to the condition and the position within the scanpath (red: short search interval, green: first fixation in the long search interval, and blue: second fixation in long search interval). Dots depict mean fixation locations aggregated for each subject individually, error bars show the standard deviation for the fixation location aggregated over all data. The scanpaths suggested by the best fitting models for the planning observer and the myopic observer are shown in the center and the right column, respectively. Again, solid lines depict the strategy for the long search interval, dashed lines for the short search interval. Global means of the human data are also shown for reference (red, green, and blue). (**b**) Actual and predicted spatial relation of first saccades for all four shapes. Graphs are centered at the fixation location in the short search interval condition. Arrows depict the displacement of the first fixation location in the long search interval relative to the short interval. Arrow color corresponds to the data source. For the myopic observer, the first fixation location is the same for both conditions (indicated by the square centered at (0, 0)). (**c**) Difference in BIC between all tested models. The lower bound corresponds to a model directly estimating the mean fixation locations for each shape and condition from the data.

The computational models were utilized to automatically generate a variety of shapes such that four stimuli could be selected to maximize the discriminative power of the subsequent experiments. As shown in Fig. 2b, two of the four shapes were predicted by our models to elicit indistinguishable first fixation locations whereas two other shapes were predicted to result in different first fixation locations for the myopic and planning observers (see Methods). The mean fixation location for each participant separately for all shapes and conditions is shown in the left panel of Fig. 3a. To test whether eye movements were planned, we compared the first fixation location in the short condition to the first fixation location in the long condition for all shapes in accordance with hypothesis *H*_1_. If subjects are capable of performing planning, we expected a difference in the first fixation location for Shape S3 and S4 (*H*_3_). We used the Hotelling’s T-test to compare the bivariate landing positions of the first saccade between the two search intervals (Supplementary Table 1). Indeed, mean target locations for the first saccade were different in Shape S3 and S4. No significant differences, however, were found in shapes S1 and S2. These results are in agreement with our computational models of the myopic and planning observers.

Visual inspection suggests, that the behavioral data is closer resembled by the results of the planning observer (*H*_2_). Indeed, only the planning observer but not the myopic observer predicted a difference in fixation locations between the short and long search interval conditions. Furthermore, the direction of the spatial difference of the first fixation location between the search interval conditions followed the course suggested by our planning observer (Fig. 3b). Because the magnitude of the human spatial difference of the first fixation location was slightly smaller than the magnitude predicted by the planning observer, we extended both the myopic observer as well as the planning observer based on known facts about the visual system. The additional modeling components yielded progressively more realistic models of human visual search behavior by incorporating biological constraints leading to a bounded actor (see Methods). Specifically, we included additive costs for longer saccade amplitude (as they lead to longer scanpath durations^24^ and higher endpoint variability^25^, which humans have been shown to minimize^26^), used foveated versions of the shapes to account for the decline of visual acuity in peripheral vision^27^, and accounted for the often reported fact, that human saccades undershoot their target^28,29^.

To obtain a quantitative evaluation of the computational models, we employed model selection using the Bayesian information criterion (BIC). The two free parameters in the models, i.e. the magnitude of additive costs for saccade length and the magnitude of the undershot, were estimated using Maximum Likelihood with bivariate Gaussian error terms on subjects’ empirical data. We also estimated the covariance matrices for the models’ predictions and the behavioral gaze data to compute the BIC for each model. Figure 3c shows the difference in BIC of all models compared to the best model. The lower bound was derived by computing the mean fixation locations directly from the data for each of the four shapes as well as for each of the three fixation locations. The difference in BIC values between two models is an approximation for the log-Bayes factor and a difference Δ*BIC* > 4.6 is considered to be decisive^30^. Results clearly favor the planning observer over the myopic observer (Δ*BIC* = 139). Crucially, the planning observer without any parameter fitting still provided a better description of our human data than the myopic observer with all extensions (Δ*BIC* = 59). Further, costs for saccade amplitudes and foveation did not only improve our model fit for the planning observer but were also favored by model selection, suggesting that they are needed for better describing the eye movement data in our experiment. For the saccadic undershot model comparison was less decisive but still in favor of the full model (Δ*BIC* = 3 between planning observer with all extensions and planning observer without undershot). We also applied the MAP searcher (see Supplementary Fig. 5) but the predictions deviated severely from the data we observed.

Parameter estimates for the saccadic undershot were similar for the myopic observer (2.9%) and the planning observer (3.2%). The influence of the costs for longer saccades was higher for the myopic observer (0.69 DP/Deg) compared to the planning observer (0.34 DP/Deg). The unit of the costs is detection performance (DP in %) per degree (Deg) and states, how much performance subjects were willing to give up to shorten saccade amplitudes by one visual degree. It is important to note, that both factors, costs and saccadic undershot, represent distinct computational concepts. The influence of the costs does not depend on the amplitude of the saccade directly, but on the reward structure of different potential landing locations. Hence, for two different shapes the same costs can have very different effects on where to target gaze. On the other hand, the undershot is relative to and only depends on the amplitude of the saccade and does not depend on the reward structure and therefore the shape. We also estimated the radius of the circular gaze contingent search shape centered at the current fixation. Parameter estimation yielded values very close to the true radius and did not improve model quality for neither the planning observer nor the myopic observer.

## Discussion

Considerable previous research has investigated perceptual determinants of human gaze targets but much less is known about how the visual system uses perceptual beliefs to select gaze targets in sequential behavior. Thus, it has been unclear, whether sequences of human eye movements are planned ahead. Prior studies indicated that multiple saccadic targets are jointly prepared as a scanpath and that cueing new targets during execution of eye movements results in longer execution times^21–23^. However, it has been unclear whether eye movements are chosen by considering more than a single gaze shift ahead into the future. Instead, paradigms modeling human eye movements as sequential greedy decisions^10,12,18,19^ including the MAP searcher^13^ and the ideal searcher^8^, have been the predominant approach. Computationally, if a task requires multiple gaze shifts in sequence, the normative, i.e. optimal solution in general involves planning the sequence of gaze shifts jointly.

The present study investigated, whether human gaze shifts are well described by a greedy selection of targets or whether they are better described by a probabilistic planning strategy. Therefore, we contrasted a myopic observer with a planning observer that was formalized within the framework of Markov Decision Processes^15,16^ with partially observable states^17^. We derived policies for myopic observers including the ‘maximum-a-posteriori searcher’^13^ and the ideal-observer based searcher^8^, which only consider the immediate reward for action selection, and we also derived the policy for the planning observer, which also considers future rewards. Next, we determined the specific circumstances under which the models produce different gaze sequences. Ultimately, we used these insights to automatically manufacture stimuli that maximized the behavioral differences elicited by the different gaze strategies and also obtained stimuli that show very similar strategies. Thus, the resulting stimuli were highly suitable for examining which gaze strategy was adopted by our subjects.

We developed a visual search task where we expected different behavioral sequences depending on the gaze strategy of our subjects. In particular, we investigated whether subjects adjust their scanpath during visual search dependent on the duration of the search interval. Therefore, we controlled the length of the saccadic sequence. The short search interval allowed subjects to execute a single saccade, while in the long search interval subjects were able to fixate two locations. The gaze contingent paradigm allowed efficient computation of all strategies including the planning strategy of gaze targets. Moreover, the gaze contingent paradigm with a search interval allowing for two saccades provides the possibility for comparing spatial gaze targets, as the well known interindividual and intradindividual variability of gaze targets for longer gaze sequences would render such comparisons computationally very difficult.

Our results suggest that eye movements are indeed planned according to probabilistic planning. Subjects’ scanpath was very well predicted by the planning observer while showing severe deviations from the scanpath proposed by the myopic observer. We found fixation locations to be different depending on the duration of the search interval. This difference is only expected under the planning observer and can not be explained by the myopic observer. Finally, model comparison favored the planning observer and its extensions over the myopic observer by a large margin. Furthermore, extending our planning observer model with action costs, we found evidence that subjects traded off task performance and saccade amplitude. Including additive costs for saccades with great amplitude into the planning observer and accounting for saccadic undershot and foveation was best capable of explaining our data further.

A possible limitation of the current experiments lies in the specific use of the gaze contingent experimental paradigm. Considerable previous research has utilized gaze contingent setups^31^ and some of these investigations have quantified its influence on performance in visual search for targets with low visibility^32^. In the present experiments, the target was only detectable within a circular area with a diameter of 13° of visual angle around the fixation point. While peripheral processing was not impaired, as the contour of the shape was always visible, the visibility of the target was controlled by the gaze contingent design. This is different from naturalistic search tasks. While this may not affect the target of the first fixation, it is conceivable that this may have affected the selection of the second gaze target in idiosynchratic ways. However, the current quantitative analyses are all based on trials in which the target was not present. Thus, the peripheral visual information acquired during the first fixation would not have given indications of the target’s position, even with visibility extending beyond 13° of visual angle because of the high visibility of targets used in our search task. Nevertheless, future work needs to address, whether the results reported here extend to experimental paradigms with full peripheral visibility.

The current experiments also do not speak to the applicability of the probabilistic planning model to other search tasks or more naturalistic visual and visuomotor tasks^3,33^. Computationally, probabilistic planning is the optimal solution to control tasks with uncertainty in general^15,16^ and evidence for human motor behavior being explainable in these terms has been provided in the past^17,26,34^. But it is currently unclear, whether the planning used in the current study can also explain sequential behavior in other visual and visuomotor tasks. Potentially, subjects may have adopted a gaze target strategy which was particularly elicited by the current experimental setup. Note however, that subjects readily adopted the reported strategy without extensive practice. Only about a minute of familiarization with the gaze contingent setup was sufficient for subjects to target gaze at the locations predicted by the planning observer model. Overall, it is an empirical question for future work whether the probabilistic planning model reported here is able to successfully account for human gaze behavior in other visuomtor tasks.

A further limitation of the current study is that it does not disambiguate between open-loop and closed-loop planning^15^. The distinction between these two types of planning lies in the way future observations are utilized within the planning process. While open-loop algorithms plan a sequence of actions but disregard the outcome of future observations, closed-loop algorithms are much more sophisticated by taking all possible future observations after each action in the entire sequence into account within the planning process. As such, closed-loop planning is even more demanding computationally than open-loop planning. The current experiments cannot disambiguate whether human behavior is better explained by either of these two planning algorithms, because for the second fixation in the long search interval condition the belief after the first fixation only depends on whether the target was found. Thus, subjects terminating the search in the long search interval condition after finding the target after the first fixation may be the only support for closed-loop control in our experiments. Note that both these planning algorithms are very different from myopic action selection and the MAP searcher. Future work will need to address, which of these two types of planning better describes human gaze selection.

Finding and executing near optimal gaze sequences is crucial for many extended sequential every-day tasks^3,33^. The capability of humans to plan behavioral sequences gives further insights into why we can solve so many tasks with ease, which are extremely difficult from a computational perspective. In many visuomotor tasks coordinated action sequences are needed rather than single isolated actions^35^. This leads to delayed rewards and thus a complex policy is required rather than an action that directly maximizes the performance after the next single gaze switch. Additionally, our findings have implications for future models of human eye movements. While numerous influential past models have not taken planning into consideration^8,10,11,14,18^, our results indicate that in the case of visual search humans are capable of including future states into the selection of a suitable scan path. Thus, perception and action are not repeatedly carried out sequentially but intertwined through planning.

Nevertheless, our results also open up the possibility to reevaluate previous studies, which have interpreted deviations from an ideal observer based search strategy as evidence for a suboptimal strategy^36–39^. The current study points towards a potential explanation of these results, as subjects may have carried out a planning strategy, which can differ from a myopic ideal observer based strategy. Given that for some stimuli in our search task the gaze sequences for the two strategies differ, future work needs to carefully reevaluate myopic and planning strategies for these tasks and stimuli, in which suboptimality was established with respect to a myopic ideal observer model. The current results furthermore suggest, that this reevaluation may need to be extend to previous studies, which have interpreted behavioral results as support for a myopic strategy^8,10–14,18,19^, as for some tasks and stimuli, the strategies lead to indistinguishable gaze targets.

The broader significance of the present results beyond the understanding of eye movements lies in the fact that human behavior in our experiment was best described by a computational model that implements probabilistic planning under perceptual uncertainty and accounts for multiple costs. In this framework, sensory measurements and goal directed actions are inseparably intertwined^40,41^. So far, the predominant approach to probabilistic models in perception has been the ideal observer^42,43^, which can be formalized in the Bayesian framework^44,45^ as inferring latent causes in the environment giving rise to sensory observations. Models of eye movements selection have so far used ideal observers^8,10,11^ without planning. Probabilistic, Bayesian formulations of optimality in perceptual tasks^46,47^, cognitive tasks^48,49^, reasoning^50^, motorcontrol^34^, learning^51^, and planning^52^ have lead to a better understanding of human behavior and the quest to unravel, how the brain could implement these computations^53–55^, which are known in general to be intractable^56^. Our result extends the current understanding by demonstrating that planning under perceptual uncertainty is also part of the repertoire of human visual behaviors and this opens up the possibility to understand recent neurophysiological results^57^ within the framework of planning under uncertainty.

In the current work, we applied the computational concept of planning drawn from the field of AI to the literature of empirical eye movement studies. In particular, we connected the experimental paradigm of visual search to a solid mathematical foundation and for the first time systematically studied the very general connection between delayed rewards, horizon, and action selection in human eye movements. Overall, we layed out the groundwork that instantly reveals several clear implication for future studies (1) to investigate eye movement planning in different tasks, (2) to study the extent of the human planning capabilities, and (3) to revisit classic influential models as they may work only in a subset of situations.

## Methods

### Participants

Overall, 16 subjects (6 female) participated in the experiment. The subjects’ age ranged from 18 to 30 years (*M* = 21.8, *SD* = 3.1). Participants either received monetary compensation or course credit for participation. All subjects had normal or corrected to normal vision (four wore contact lenses). One subject stated to have dyschromatopsia, which had no influence on the experiment. Sufficient eye tracking quality was ensured for all data entering the analysis. In each trial a single fixation location (short search interval) or a sequence of two fixation locations (long search interval) entered the analysis. Further, informed consent was obtained from all participants, all experimental procedures were approved by the ethics committee of the Darmstadt University of Technology, and all methods employed were in accordance with the guidelines provided by the German Psychological Association (DGPs).

### Procedure

After signing a consent form the eye tracker (SMI Red, 250 Hz) was calibrated using a 3 point calibration procedure. Subsequently, subjects completed three to five short training trials (about 1 minute duration in total) as part of the experiment instruction. During these training trials it was ensured that the search time was sufficiently long for each subject to carry out a single saccade in the short condition and two fixations in the long condition, respectively. If necessary, the search time was adjusted (between 500 ms and 580 ms, for the long search interval). Participants were encouraged to ask questions, if anything was unclear. After training, participants answered ten questions from a checklist to ensure that they understood the task properly (e.g.: “When does the search interval start and how many targets can be found at most?”). Incorrect answers were documented and the correct answers were discussed. After successfully finishing the training, four blocks each containing 100 trials were performed. Thereby, the order of the blocks was either SSLL (two blocks with short search time followed by two blocks with long search time) or LLSS. Participants were randomly assigned to one of the two orders. Participants’ heads were supported using a chin rest leading to a constant viewing distance of 55 cm. Eye tracking calibration was renewed before each block. Trials in which the first saccade was made while the fixation cross was still visible were dismissed and had to be repeated.

### Materials

The derived and implemented computational models enabled us to specifically select shapes that facilitate testing our hypothesis. In particular, stimuli were identified that triggered different policies for the myopic observer and planning observer. First, multiple candidates shapes were generated automatically using the following approach: Five points were drawn uniformly in a bounded area (23.24 × 23.24 of visual angle). Next, a B-spline was fitted to the random points. Finally, the shapes bounded by the splines using the fitted parameters were filled with a white noise texture. Both models were applied to the resulting shapes to identify those shapes that lead to maximally different or most similar policies. Overall, four different shapes were used in the experiment (see Fig. 2b). Two shapes were chosen where optimal behavior requires planning (S3 and S4) and two where planning is not necessary (S1 and S2), i.e. where the sequence of eye movements of the myopic observer and the planning observer coincide. For both categories we selected two shapes by visual inspection ensuring that they were similar with respect to the area covered. For display during the experiment the shapes were upscaled with a factor of 1.5 and centered on the monitor such that the center of the shape’s bounding box matched the center of the screen.

### Foveated versions of the stimuli

In order to account for the decline of visual acuity that affects the visibility of the shape boundaries, we created foveated versions of the experimental stimuli (see Supplementary Fig. 4). Foveated versions of the stimuli were created by using the approach described in ref.^58^. The contrast sensitivity function describing the decline of accuracy with increasing eccentricity is computed as

4 $$CS(f,e)=\frac{1}{CT(f,e)},$$

which can be used to assign a cut-off frequency at each eccentricity. The contrast threshold is computed as:

5 $$CT(f,e)=C{T}_{0}\,\exp (\alpha f\frac{e+{e}_{2}}{{e}_{2}}).$$

where *f* is the spatial frequency, *e* is the retinal eccentricity, and *α*, *CT*_0_, *e*_2_ are empirical values set to 0.106, 1/75, and 2.3, respectively. These values have been shown to provide a good fit to empirical data (see ref.^27^). We only needed to account for peripheral vision at the level of deciding where to make an eye movement to. The decline of visual acuity leads to deteriorated perception of the outline of the shape. Hence, our model needs to incorporate the foveated shapes. For the first fixation, we used the initial location at the beginning of the trial. This was the same for all trials. In the two saccade condition we used the empirical mean landing location of our participants to compute the foveated shape prior to the second saccade. While this is an approximation as landing locations showed variation, we did so to reduce the computational burden of the numerical optimization.

The target was a circular grating stimulus (0.87° of visual angle in diameter). Background was Gaussian white noise. Contrast was set in a way that the target was easily detected if it was within the visible search radius of the current fixation (detection proportion: 98.2%). The target’s position was generated by randomly choosing a location within the shape.

### Probability of finding the target

Next, we derive the probability of a correct detection given a sequence of fixation locations since both proposed policies depend on the performance in the task, i.e., the detection probability. The probability of correctly judging the presence of a target is proportional to the area covered by the search. This can be computed as:

6 $$P({\rm{correct}}|{x}_{n},{y}_{n})\propto \sum _{x}\sum _{y}\,{P}_{T}(x,y){P}_{O}(x,y|{x}_{n},{y}_{n})$$

where *P*_T_(*x*, *y*) is the probability that the target is located at (*x*, *y*) and *P*_*O*_(*x*, *y*|*x*_*n*_, *y*_*n*_) is the probability that the location (*x*, *y*) is covered by the search given that the saccade was targeted at (*x*_*n*_, *y*_*n*_). The former is 1/*N* if (*x*, *y*) lies within the shape and zero otherwise, where *N* is the number of possible target locations. The latter depends on the distance between the saccadic target (*x*_*n*_, *y*_*n*_) and the target location (*x*, *y*). Therefore:

7 $${P}_{O}(x,y|{x}_{n},{y}_{n})=(\begin{array}{ll}1 & {\rm{if}}\,\parallel {[{x}_{n}-x,{y}_{n}-y]}^{T}\parallel  < {\rm{threshold}}\\ 0 & else\end{array}$$

where the threshold is equal to the radius of the search area (6.5° of visual angle).

### Perception

Visual perception can be described as inference of latent causes based on sensory signals^44,45^. Bayesian inference provides the mathematical tools to use sensory data *D* to infer unknown properties of the state *s* of the environment. For example, *s* could be indicating whether there is a predator hiding behind a bush, and by directing gaze to the bush visual data *D* about the latent variable describing the true state *s* of the environment is obtained. This information can be incorporated into what is known about *s* using Bayes’ theorem $P(s|D)=P(D|s)P(s)/P(D)$. Hence, this mechanism combines prior knowledge *P*(*s*) and sensory information *P*(*D*|*s*) to form an updated posterior belief about environmental states relevant to the specific task.

### Action

However, performing sensory inference by itself does not prescribe an action, i.e. information about *s* in the end needs to be used to decide for an appropriate action, e.g. whether to flee. The costs and benefits for the potential outcomes of the action can be very different, e.g., not to flee if a predator is present is more costly than an unnecessary flight. They can be captured computationally by a reward function *R*(*s*, *a*) assigning a value to each state action pair. In the past, different approaches have been proposed to choose an action given the current belief *b*(*s*) = *P*(*s*|*D*) drawn from perception and the reward function *R*(*s*, *a*).

*The MAP model* only takes into account the maximum of the posterior for action selection. This corresponds to taking the action

8 $${\pi }_{{\rm{MAP}}}={\rm{\arg }}\,\mathop{{\rm{\max }}}\limits_{a}\,R(a,s={\rm{\arg }}\,\mathop{max}\limits_{s}\,P(s|D))$$

that gives maximum reward given that the true state is the maximum of the posterior. For visual search arg max_*s*_ *P*(*s*|*D*) corresponds to the most likely location of the target and therefore the reward is maximal if the eye movement *a* is targeted towards this location.

*The ideal observer model* has been used successfully to understand how humans choose locations for the next saccade. Specifically, human eye movements use the current posterior and target the location where they expect uncertainty about task relevant variables to be reduced most after having acquired new data from that location in situations such as visual search^8^, face recognition^10^, and temporal event detection^11^. Hence, different potential outcomes of *s* are weighted with the reward function *R*(*s*, *a*) to determine the action with highest expected reward:

9 $${\pi }_{{\rm{i}}{\rm{d}}{\rm{e}}{\rm{a}}{\rm{l}}{\rm{o}}{\rm{b}}{\rm{s}}{\rm{e}}{\rm{r}}{\rm{v}}{\rm{e}}{\rm{r}}}=\arg \,\mathop{max}\limits_{a}\,{\mathbb{E}}[{r}_{0}]=\arg \mathop{max}\limits_{a}{\int }_{s}R(a,s)P(s|D)ds.$$

Thus, it may be better to flee, even when one is not absolutely certain that a predator is hiding behind a bush, because the consequences may be particularly harmful. Interestingly, within this framework, the optimal action targets the location where the next fixation will reduce uncertainty the most and not the location that currently looks like the most probable target location. Indeed, both explicit monetary rewards^19^ and implicit behavioral costs^11^ in experimental settings have been shown to influence eye movement choices.

*The ideal planner model* extends the ideal observer model to action sequences. While ideal observers based on Bayesian decision theory constitute the optimal solution to selecting a single action, repeatedly taking the action with the maximum immediate reward may fail in tasks with longer action sequences and delayed rewards depending on the specific task structure. In these cases, a planning observer based on the more powerful framework of belief MDPs, which contains the ideal observer as special case, is needed to find the optimal strategy. A Markov Decision Process (MDP)^16,59^ is a tuple (*S*, *A*, *T*, *R*, *γ*), where *S* is a set of states, *A* is a set of actions, $T=P(s^{\prime} |s,a)$ contains the probabilities of transitioning from one state to another, *R* represents the reward, and finally, *γ* denotes the discount factor. In a belief MDP only partial information about the current state *s* is available, therefore a probability distribution over states is kept as a belief state $b(s)=P(s|D)$^17^. The expected reward associated with performing action *a* in a belief state *b*(*s*) is denoted by the action-value function $Q(b(s),a)$. How should the actor decide where to look next according to this framework? A policy *π* is a sequence of actions and the optimal policy π^*^ comprises actions that maximize the expected reward

10 $$\begin{array}{rcl}{\pi }_{{\rm{ideal}}{\rm{planner}}} & = & {\rm{\arg }}\,\mathop{{\rm{\max }}}\limits_{a}\,{\mathbb{E}}[{r}_{0}+\gamma {r}_{1}+\ldots +{\gamma }^{n}{r}_{n}]={\rm{\arg }}\,\mathop{{\rm{\max }}}\limits_{a}\,Q(b(s),a)\\  & = & {\rm{\arg }}\,\mathop{{\rm{\max }}}\limits_{a}{\int }_{b(s^{\prime} )}\{P(b(s^{\prime} )|b(s),a)[R(b(s^{\prime} ))+\gamma {V}^{\ast }(b(s^{\prime} ))]\}db(s^{\prime} \mathrm{).}\end{array}$$

where V^*^ (*b*(*s*′)) is the expected future reward gained from the next belief state *b*(*s*′). In tasks comprising sequences of actions, the optimal strategy, the planning observer, incorporates rewards associated with future actions (V^*^ (*b*(*s*′))) into action selection. Essentially, what this means is that the value of an action based on the current belief is a combination of the immediate reward and the long term expected reward, weighted by how likely the next belief is under the action. Thus, as the belief about the state of task relevant quantities depends on uncertain observations, actions are influenced both by obtaining rewards and obtaining more evidence about the state of the environment.

### Action selection in visual search

To apply the different models to our visual search task we first need to specify the relevant quantities describing the task, i.e. the state representation and the reward function. In our visual search task (Fig. 1), a suitable candidate for a state representation is the target location and the current location of gaze. However, in general, the exact location of the target is unknown. Instead, we formalize the probability distribution over potential target location as a belief state that can be inferred from observations. The action space comprises potential fixation locations and with each action we receive information about the target, update our belief and transition to the next belief state. The reward function is an intuitive mapping between the belief state, which comprises the knowledge about the location of a potential target, and the probability of finding the target.

For a sequence comprising two actions (*a*_0_, *a*_1_), the myopic observer (*horizon* = 1, repeated application of the ideal observer) selects the action with the maximum expected reward in each step

11 $${\pi }_{{\rm{myopic}}}=(\mathop{{\rm{argmax}}}\limits_{{a}_{0}}\,{\mathbb{E}}[{r}_{0}],\mathop{{\rm{argmax}}}\limits_{{a}_{1}}\,{\mathbb{E}}[{r}_{1}]),$$

which corresponds to using Equation 9 at each state of the sequence. The planning observer (*horizon* = 2) considers the total sum of rewards

12 $${\pi }_{{\rm{planned}}}^{\ast }=(\mathop{{\rm{argmax}}}\limits_{{a}_{0}}\,{\mathbb{E}}[{r}_{0}+{r}_{1}],\mathop{{\rm{argmax}}}\limits_{{a}_{1}}\,{\mathbb{E}}[{r}_{0}+{r}_{1}]).$$

which corresponds to using Equation 10 at each state of the sequence. Whether ${\pi }_{{\rm{myopic}}}$ and ${\pi }_{{\rm{planned}}}^{\ast }$ lead to the same action sequence depends on the specific nature of the task. However, in general:

13 $${\pi }_{{\rm{planned}}}^{\ast }\ne {\pi }_{{\rm{myopic}}}$$

as can be seen in Fig. 2. Ideal-observer approaches only lead to optimal actions if future rewards do not play a role, for example, if only a single action is concerned. It is apparent that for a single action the myopic observer and the planning observer lead to the same action as Equation 10 simplifies to

14 $$Q(b(s),a)={\int }_{b(s^{\prime} )}P(b(s^{\prime} )|b(s),a)R(b(s^{\prime} ),a)db(s^{\prime} )$$

where $P(b(s^{\prime} )|b(s),a)$ is the posterior over relevant quantities in the task and $R(b(s^{\prime} ),a)$ is the reward function.

### Model fitting

To take into account known cognitive and biological constraints we incorporated several well known characteristics of the human visual system. We introduced costs on the saccade amplitude thus favoring smaller eye movements, which humans have shown to do^60^. As was shown by prior research, greater amplitudes lead to higher endpoint variability^25^ and longer saccade duration^24^. It has further been shown that humans attempt to minimize endpoint variability when execution eye movements^26^. Therefore, we hypothesized that subjects show a preference for smaller saccade amplitudes. Computationally, we obtain the total reward as a combination of performance and saccade amplitude

15 $${r}_{n}(\alpha )=P({\rm{correct}}|{x}_{n},{y}_{n})-\alpha {\rm{c}}({x}_{n},{y}_{n})$$

where *c* is a linear cost function returning the amplitude of the saccade. The parameter *α* determines how much detection probability a subject is willing to give up in order to decrease saccade amplitude^11^. It was estimated from the mean fixation locations of our participants using Maximum Likelihood.

Next, the human visual system does not have access to visual content at all locations in the field of view with unlimited precision. We accounted for the decline of visual acuity at peripheral locations. Therefore, foveated versions of the shapes were generated using the known human contrast sensitivity function (see refs^8,10,27^, for example). For the first fixation foveation was computed using the initial fixation location of the trial. As it was not computationally tractable to compute foveated images corresponding to the exact location of the first landing position, we approximated it by using the mean fixation location of our subjects instead.

Finally, prior studies have shown that saccades undershot target locations^29^. Initial landing positions are closer to the start location of a saccade. The final target is reached using subsequent corrective saccades. However, in our experiment there is no visible fixation target, therefore corrective saccades might not be present. To account for that we estimated the undershot from our data.

### Preprocessing

First, fixations were extracted from the raw gaze signal using the software of the eye tracking device. Overall, 6400 trials (16 participants × 4 blocks × 100 trials per block) entered the preprocessing. 15 trials (0.23%) were dismissed because the subjects failed to target gaze towards the shape. In these trials, subjects triggered the beginning of the trial by crossing the boundary, however did not engage in visual search. While search time was adjusted to enable subjects to perform a single saccade in the short condition and two saccades in the long condition, respectively, in 17% of the trials subjects failed to do so. Since we are only interested in comparing the difference between strategies consisting of one or two targeted locations we only used the remaining 5288 trials. Next, we excluded trials where the target was present, regardless of whether it was found, leaving 2589 trials. Clearly, behavior after successfully finding the target is confounded and does no longer provide valid information about the search strategy. Also, trials in which a target was shown but not found are biased as they are more likely to occur in the context of inferior eye movement strategies.

Our analysis and our estimated model parameters rely on mean landing positions aggregated within subjects. Therefore, we need to make sure that the variation in landing positions arises due to saccadic endpoint variability or uncertainties the subject might have about the shape, but not from qualitatively different strategies. Shapes S1 and S2 consist of two separate parts, as a consequence the reward distribution is no longer unimodal across potential gaze targets (see Supplementary Fig. 1a). Indeed, qualitatively different strategies in the short condition were found for these stimuli (see Supplementary Fig. 1b). Using mean gaze locations therefore would have lead to misleading results as it implicitly implied unimodal variability in landing positions while the real data showed clear multi-modality. To further analyze the gaze targets of our participants, we first identified the strategy for each trial using a Gaussian mixture model. We only considered the most frequent strategy (see Supplementary Fig. 1c) for both shapes and discarded trials (10.6%) deviating from the chosen strategy. However, our findings do not depend on the particular choice of strategy as shapes that revealed differences between the myopic observer and the planning observer (S3 and S4) did not elicit different strategies. The remaining 2313 trials were used for our analysis.

### Code Availability Statement

The code used in this study is available from the corresponding author on request.

## Supplementary information

Supplementary Information

## Data Availability

The data that support the findings of this study are available from https://github.com/RothkopfLab/spatial_gaze_planning.
