# Supplementary material for: Multi-step planning of eye movements in visual search
Source: Sci Rep. 2019 Jan 15;9:144. doi: 10.1038/s41598-018-37536-0 (PMC6333838; doi:10.1038/s41598-018-37536-0)
Supplement: Supplementary file 1 — Supplementary Information [file 41598_2018_37536_MOESM1_ESM.pdf]

Supplementary Information

Multi-step planning of eye movements in visual search

Hoppe David[1,2], *Rothkopf Constantin A.[1,2,3]

1 Department of Psychology, Technical University Darmstadt, Darmstadt, Hesse, Germany
2 Centre for Cognitive Science, Technical University Darmstadt, Darmstadt, Hesse, Germany
3 Frankfurt Institute for Advanced Studies, Goethe University, Frankfurt, Hesse, Germany

Supplementary Table 1: Descriptive statistics of landing positions (in screen pixel) for all shapes as well as inferential statistics (two-sided) for the comparison across conditions (long and short).

		N	Centroid		Covariance			Hotelling's T	p
			$\bar{x}$	$\bar{y}$	σ^2_{xx}	σ^2_{yy}	σ^2_{xy}		
S1									
	Short	16	540	568	862	686	191	0.744	.485
	Long		535	554	1 018	1040	513		
S2									
	Short	16	669	622	267	1270	271	2.66	.086
	Long		669	650	223	1574	210		
S3									
	Short	16	766	554	1676	3991	-2136	17.43	1.07×10^{-5}*
	Long		705	675	550	3207	-492		
S4									
	Short	16	748	608	3903	815	-106	7.89	.002*
	Long		677	631	2094	1066	-223		

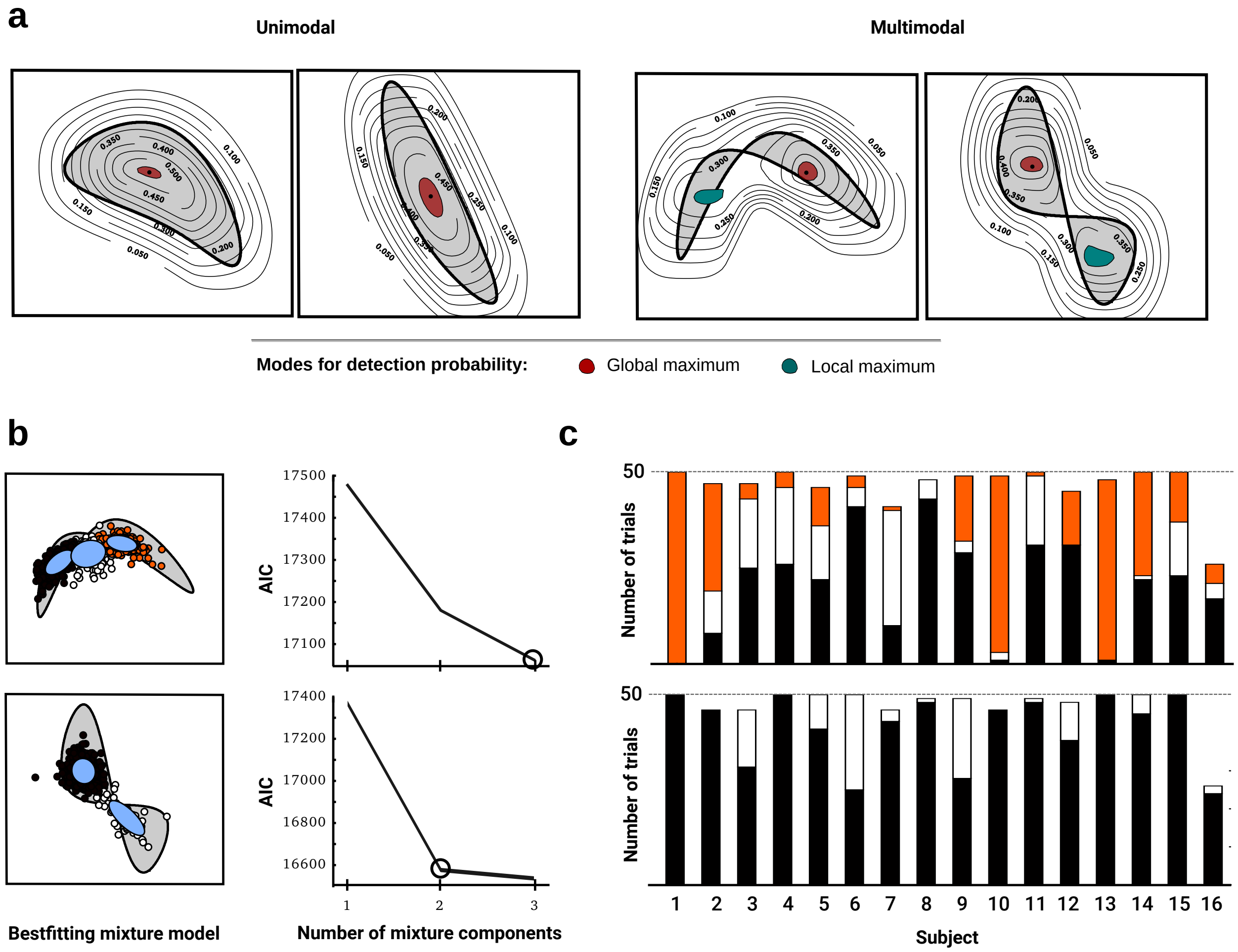

Supplementary Figure 1: (**a**) Theoretical detection performance for the shapes used in the experiment. Contour lines depict the percentage of detected events dependant on the fixation location. Two shapes show unimodal detection performance maps, two have multiple modes. (**b**) Raw data for the two multimodal shapes. Gaussian mixture models were fitted to our subjects raw data. Model comparison revealed qualitatively different strategies. First fixations were distributed over the different modes for multimodal shapes. (**c**) Proportion of trials corresponding to the different strategies. Panels are aligned to (b). Color represent the choice of strategy according to the mixture model shown in (b).

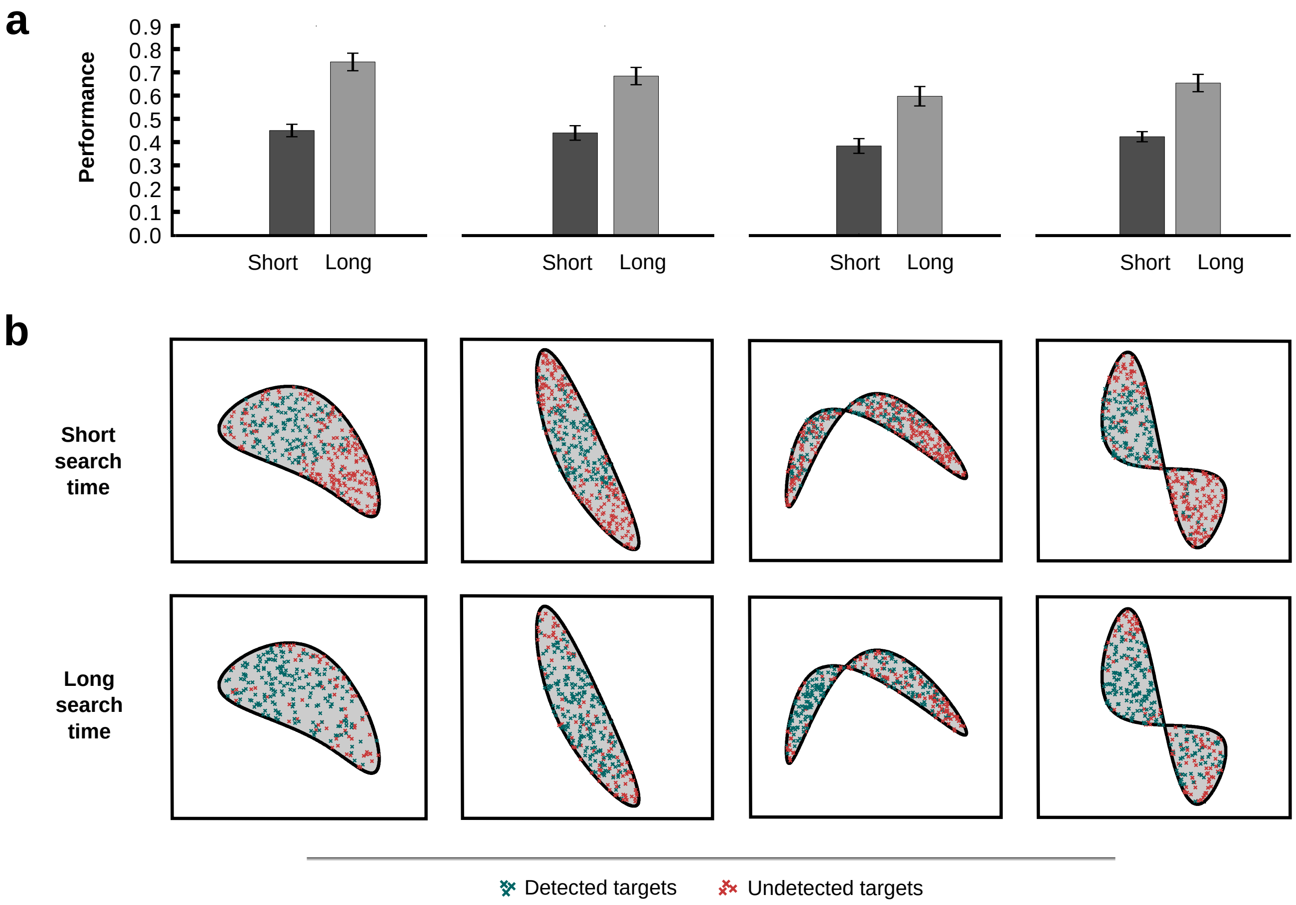

Supplementary Figure 2: (**a**) Human detection performance for all shapes and conditions separately. Mean performance over all subjects (N = 16) is shown. Error bars correspond to the standard error of the mean. (**b**) Error distribution for all four shapes and both conditions. Green crosses depict locations where a target was successfully detected. Red cross depict locations of targets that were missed. The upper panel shows the error distribution for the short condition. The errors for the long condition are shown in the lower panel.

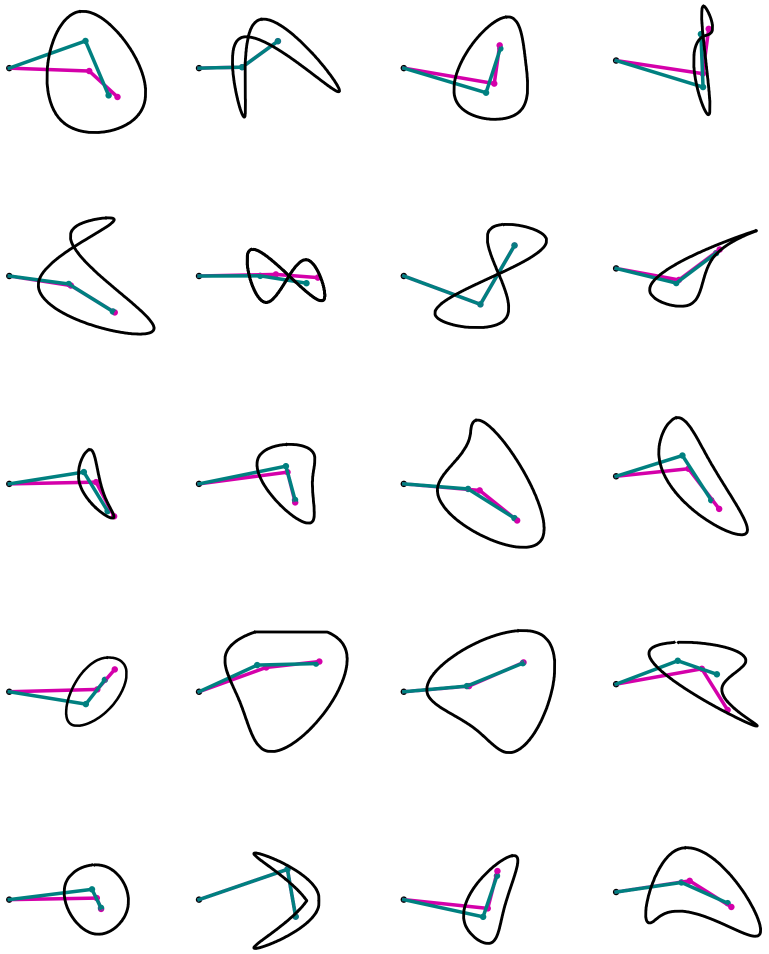

Supplementary Figure 3: Shapes generated by the algorithm with random initialization. Green and pink lines correspond to the optimal two fixation search strategy of the planning observer and the myopic observer, respectively.

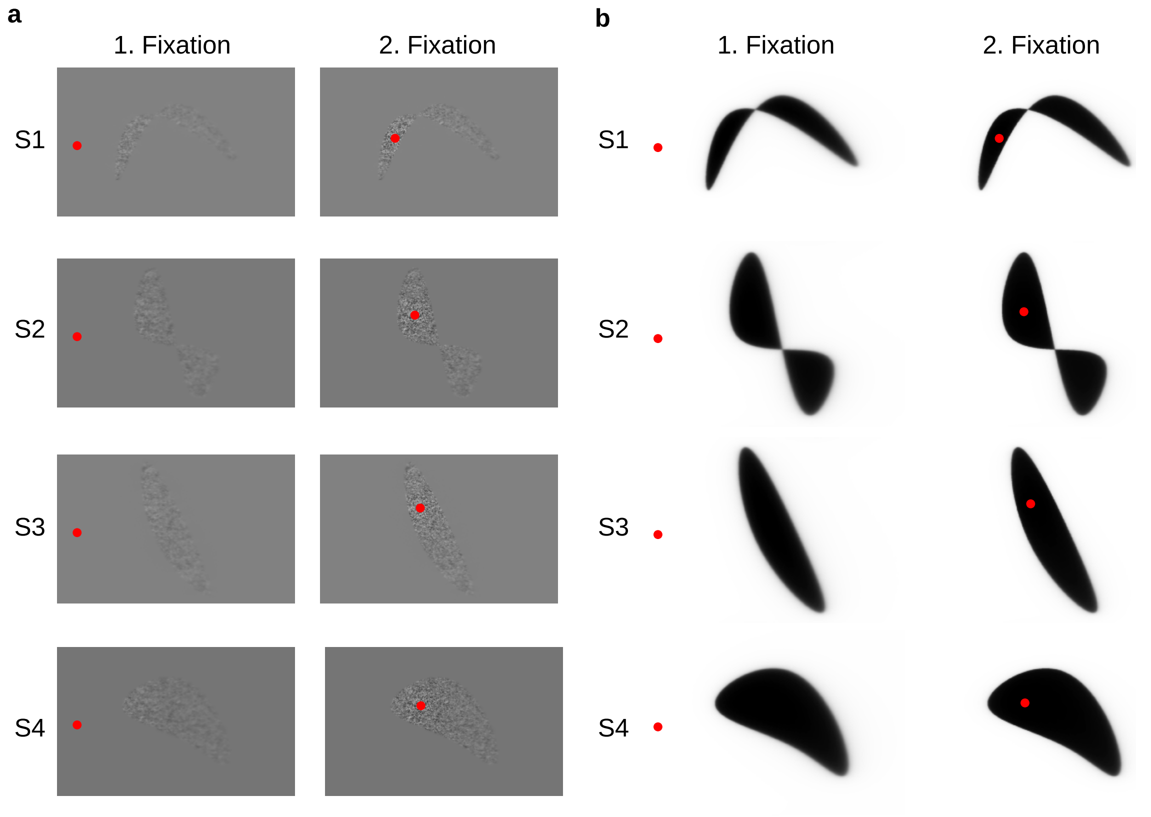

Supplementary Figure 4: Foveated versions of the experimental stimuli. (**a**) Red circles correspond to the current fixation. All shapes depict the location of the planned policy as second fixation location. Shapes are filled with white noise to illustrate the decline of details with greater distance to the fixation location. (**b**) Same as (a). Instead of texture the probability of a location being part of a shape is shown. The black area inside the shape corresponds to a high probability of being part of the shape.

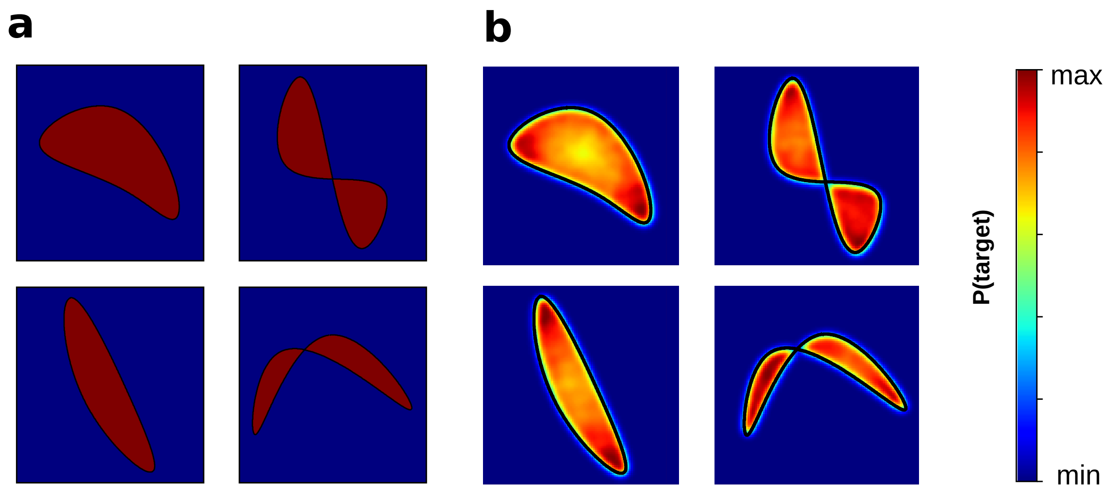

Supplementary Figure 5: Simulated eye movement density for the MAP searcher. The MAP searcher chooses the location associated with the maximum probability of containing the target (the mode of the probability distribution over potential target locations. **(a)** For the short condition in our task target distribution is uniform inside the shape. Hence, all locations are equally likely to be chosen by the MAP searcher. **(b)** For the long condition, the target probability prior to the second fixation depends on the location of the first fixation. After the first fixation, the target probability is uniformly distributed over all locations that were not covered by the fixation (further away than the search radius), which follows directly from our experimental design. Hence, the MAP searcher choses uniformly locations that have not been covered. The density depicts the data from 50 000 simulated runs of the MAP searcher. Clearly, these deviate from the eye movement data we observed from our participants.

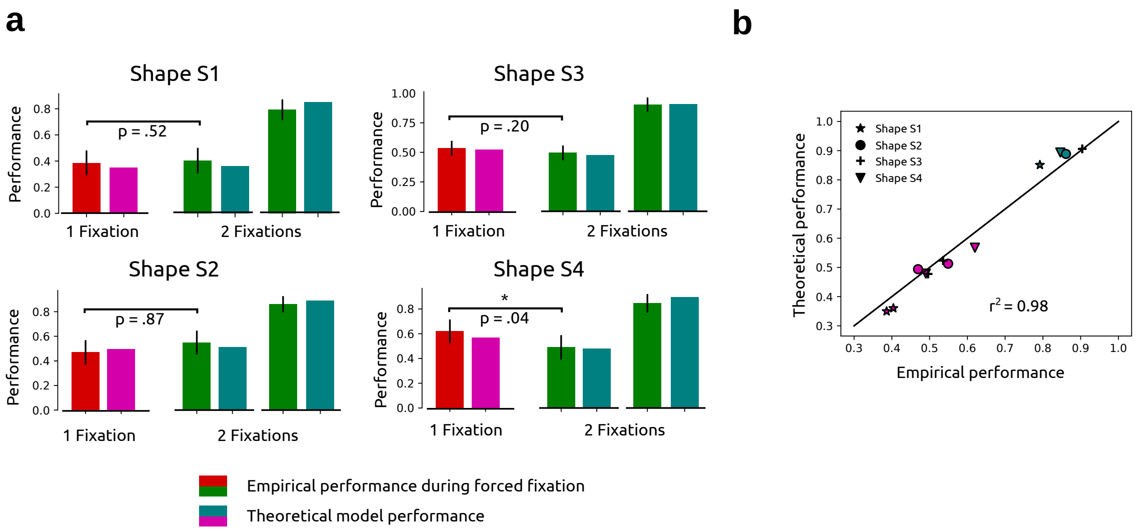

Supplementary Figure 6: Empirical performance and model performance. **(a)** We collected empirical performance data from three additional participants (two were naive to the study) as follows: Subjects executed either one or two fixations for all shapes using the mean fixation location from our main experiment. Subsequently they reported whether they had found the target. Targets were presented in every trial to accelerate estimation of mean detection performance. The influence of endpoint variability on the detection probability in the fixed fixation sequence was prevented in order to be able to compare the behavioral data to our model results (as the computational performance does not include variability). In total, 1882 additional trials were conducted. The percentage of successful trials for our subjects is depicted for the short condition (red) as well as for the long conditions (green). Performance of the scanpaths is shown after the first as well as after the second fixation. Results suggest better performance in the first fixation in the short condition for Shape S4, which is also predicted by the framework (see Fig. 2b in the main text). For Shape S3 there also was a difference, however, much more data is needed to reach significance (estimated in the range of 1000 to 1500 trials according to power analysis). **(b)** Behavioral data from the forced fixation task closely reflect the model predictions from the planned model for the performance.
